# Medium-chain fatty acids and monoglycerides as feed additives for pig production: towards gut health improvement and feed pathogen mitigation

**DOI:** 10.1186/s40104-020-00446-1

**Published:** 2020-04-23

**Authors:** Joshua A. Jackman, R. Dean Boyd, Charles C. Elrod

**Affiliations:** 1 School of Chemical Engineering, Sungkyunkwan University, Suwon, 16419 Republic of Korea; 2 Hanor Company, Franklin, KY 42134 USA; 3 North Carolina State University, Raleigh, NC 27695 USA; 4 Natural Biologics Inc., Newfield, NY 14867 USA; 5 Department of Animal Science, Cornell University, Ithaca, NY 14853 USA

**Keywords:** Antibiotics, Feed pathogen mitigation, Growth promotion, Gut health, Immune enhancement, MCFA, Medium-chain fatty acids, Monoglycerides

## Abstract

Ongoing challenges in the swine industry, such as reduced access to antibiotics and virus outbreaks (e.g., porcine epidemic diarrhea virus, African swine fever virus), have prompted calls for innovative feed additives to support pig production. Medium-chain fatty acids (MCFAs) and monoglycerides have emerged as a potential option due to key molecular features and versatile functions, including inhibitory activity against viral and bacterial pathogens. In this review, we summarize recent studies examining the potential of MCFAs and monoglycerides as feed additives to improve pig gut health and to mitigate feed pathogens. The molecular properties and biological functions of MCFAs and monoglycerides are first introduced along with an overview of intervention needs at different stages of pig production. The latest progress in testing MCFAs and monoglycerides as feed additives in pig diets is then presented, and their effects on a wide range of production issues, such as growth performance, pathogenic infections, and gut health, are covered. The utilization of MCFAs and monoglycerides together with other feed additives such as organic acids and probiotics is also described, along with advances in molecular encapsulation and delivery strategies. Finally, we discuss how MCFAs and monoglycerides demonstrate potential for feed pathogen mitigation to curb disease transmission. Looking forward, we envision that MCFAs and monoglycerides may become an important class of feed additives in pig production for gut health improvement and feed pathogen mitigation.

## Introduction

Antibiotics have long been used in the swine industry for therapeutic purposes, as they are in human medicine, to prevent severe clinical disease and death [1]. Antibiotics work against bacteria and different classes of antibiotics have distinct targeting spectrums and potencies [2]. Certain antibiotics have also been and continue to be used for treating subclinical diseases [3], and successful intervention can indirectly improve pig growth and feed efficiency [4]. Mechanistically, antibiotics can affect bacterial cell integrity and reproduction, and thus exert growth-promoting effects by modulating the composition of bacterial populations within the pig gastrointestinal tract while reducing bacterial pathogen levels throughout the body to prevent disease.

Despite these important capabilities, there are mounting concerns that antibiotic usage in food animals is contributing to the global problem of antibiotic-resistant bacteria across human and animal populations [5]. These concerns have led to calls to significantly reduce or stop the use of antibiotics in animals that are also used in human medicine, particularly in cases where they are used at sub-therapeutic levels to promote growth [6]. Legislative bans on growth-promoting antibiotics have been passed in different parts of the world, such as the European Union [7], while other regulatory actions, such as the revised Veterinary Feed Directive in the United States, are encouraging more judicious use of human medically relevant, therapeutic antibiotics in food animal production, including halting sub-therapeutic usage of such antibiotics [8]. At the same time, discussion has been raised about how curtailing the use of growth-promoting antibiotics that act as prophylactics to inhibit bacterial pathogens at the subclinical level could lead to the increased use of therapeutic antibiotics among pigs displaying clinical infections [9].

Concerns and legislative actions around antibiotic use in food animals are leading to an increase in the search for tools to counter pathogens. Promoting immune development or expression in young animals is one example, which also tends to improve growth [10]. A recent addition to vaccines, therapeutic drugs, and immune enhancers is medium-chain fatty acids (MCFAs), especially saturated MCFAs with 6–12 carbon-long chains, which have demonstrated positive benefits as feed additives by improving animal health, production, and feed digestibility [11]. These efforts have built on pioneering works that utilized medium-chain triglycerides (MCTs) as feed additives in conjunction with lipolytic enzymes, called lipases, that can catalyze *in vivo* MCT hydrolysis and subsequent release of biologically active MCFAs and monoglyceride derivatives to support pig growth and development [12–15]. Importantly, free MCFAs and monoglycerides also exhibit antiviral and antibacterial activity [16, 17]. The combined health-promoting and pathogen-mitigating functions of MCFAs and monoglycerides are particularly significant in light of the expanding African Swine Fever virus (ASFv) epidemic, which highlights the devastation of, and urgency to address, virus outbreaks in the swine industry [18]. Over the past few years, there has been progress in evaluating the performance of free MCFAs and monoglycerides as feed additives in pig diets as well as in understanding mechanistic aspects of how these compounds function. MCFAs and their respective monoglycerides have also proven capable of mitigating some feedborne pathogens.

The objective of this review article is to summarize the latest advances in the use of MCFAs and monoglycerides as health-promoting feed additives and feed pathogen mitigation agents for pig production. This article extends beyond previous reviews that broadly surveyed the health-promoting effects of different types of fatty acids [19, 20], while providing more recent and deeper coverage of the chemical properties, pathogen disruption mechanisms, and field tests of MCFAs and monoglycerides; the latter being an important class of MCFA derivatives that has not been reviewed before [21, 22]. In addition, we provide coverage of MCFAs and monoglycerides as feed pathogen mitigation agents and discuss current progress to inhibit high-priority viruses in feed, which is significant because feed can be an important vector for transboundary diseases [23]. We also address MCFA and monoglyceride encapsulation and delivery in a variety of formats that may be useful for feed pathogen mitigation, for enteric pathogen mitigation, and for delivery to distant enteric sites.

## Medium-chain fatty acids and monoglycerides

MCFAs (defined as saturated fatty acids with C_6_ to C_12_ hydrocarbon chains) and their corresponding monoglyceride derivatives are single-chain lipid amphiphiles. An overview of the basic physical properties of important MCFAs and monoglycerides is presented in Table 1.

**Table 1:** Overview of medium-chain fatty acids and monoglycerides

	Compound name	Chemical structure	Molecular weight, Da	Melting point, °C	Visual appearance	Smell
Fatty Acids	Caproic acid (C_6_H_12_O_2_)		116.2	− 3.4	Oily colorless liquid	Strong
	Caprylic acid (C_8_H_16_O_2_)		144.2	16.5	Oily colorless liquid	Moderate
	Capric acid (C_10_H_20_O_2_)		172.3	31.6	White crystalline powder	Mild
	Lauric acid (C_12_H_24_O_2_)		200.3	43.8	White powder	Minor
Monoglycerides	Glycerol monocaproate / Monocaproin (C_9_H_18_O_4_)		190.2	19.4	Colorless liquid	Minor
	Glycerol monocaprylate / Monocaprylin (C_11_H_22_O_4_)		218.3	35.6	White powder	Minor
	Glycerol monocaprate / Monocaprin (C_13_H_26_O_4_)		246.3	51.4	White powder	Minor
	Glycerol monolaurate / Monolaurin (C_15_H_30_O_4_)		274.4	62.5	White granular powder	Minor

The smell level is ranked in the order of strong, moderate, mild, and minor. The molecular formula of each compound is provided in parentheses and the chemical structures of MCFAs are drawn in the deprotonated state.

MCFAs and monoglycerides are naturally present in the milk produced by some animals [24] as well as in other natural sources such as coconut oil [25]. They are building blocks of MCTs, which can be used as feed additives and lipases break down the triglycerides *in vivo* to yield MCFAs and monoglycerides among various hydrolysis products [13, 15]. The MCT approach is useful because, unlike certain MCFAs, they do not have adverse odors and facilitate digestion and release of MCFAs and monoglycerides in the stomach along with progressive release in the foregut as well. Depending on the situation, the MCTs may be delivered as a standalone feed additive if the feed source contains endogenous lipases or if gastric lipase secretion in the pig is sufficiently developed, or can be delivered together with an exogenous lipase [14].

Another option that is becoming popular and is the main focus of this review article involves directly using high-purity forms of desired MCFAs and/or monoglycerides as the additive, and to directly incorporate them into feed and/or water. An important benefit of directly using free MCFAs and/or monoglycerides in feed is that they exhibit antimicrobial properties and thus can potentially inhibit viral and bacterial pathogens in the feed to reduce the risk of disease transmission. Of note, depending on the jurisdiction, MCTs, MCFAs, and monoglycerides may also be classified as feed materials instead of as feed additives, which allows them to be incorporated into animal feed without registration. A brief description of the chemical structures, membrane-disruptive activities, and related biological activities of MCFAs and monoglycerides is provided in this section.

### Chemical structure

Fatty acids are hydrocarbon chains wherein one end of the hydrocarbon chain has a carboxylic acid functional group with a pK_a_ value around pH 5. Most fatty acid molecules are anionic (deprotonated) around neutral pH conditions, while they are mainly nonionic (protonated) in acidic pH environments such as the stomach. Fatty acids with saturated hydrocarbon chains are generally preferable to work with because saturated fatty acids are more chemically stable and less prone to oxidation-related rancidity [26]. Medium-chain saturated fatty acids are of particular interest because of their reported antimicrobial activity. Monoglycerides of MCFAs are another type of single-chain lipid amphiphile that is comprised of esterified adducts of one fatty acid molecule and one glycerol molecule. The hydroxyl groups of monoglycerides have very high pK_a_ values (around 14), thereby remaining nonionic across physiologically relevant pH conditions.

### Mechanism of action

**Fig. 1:**
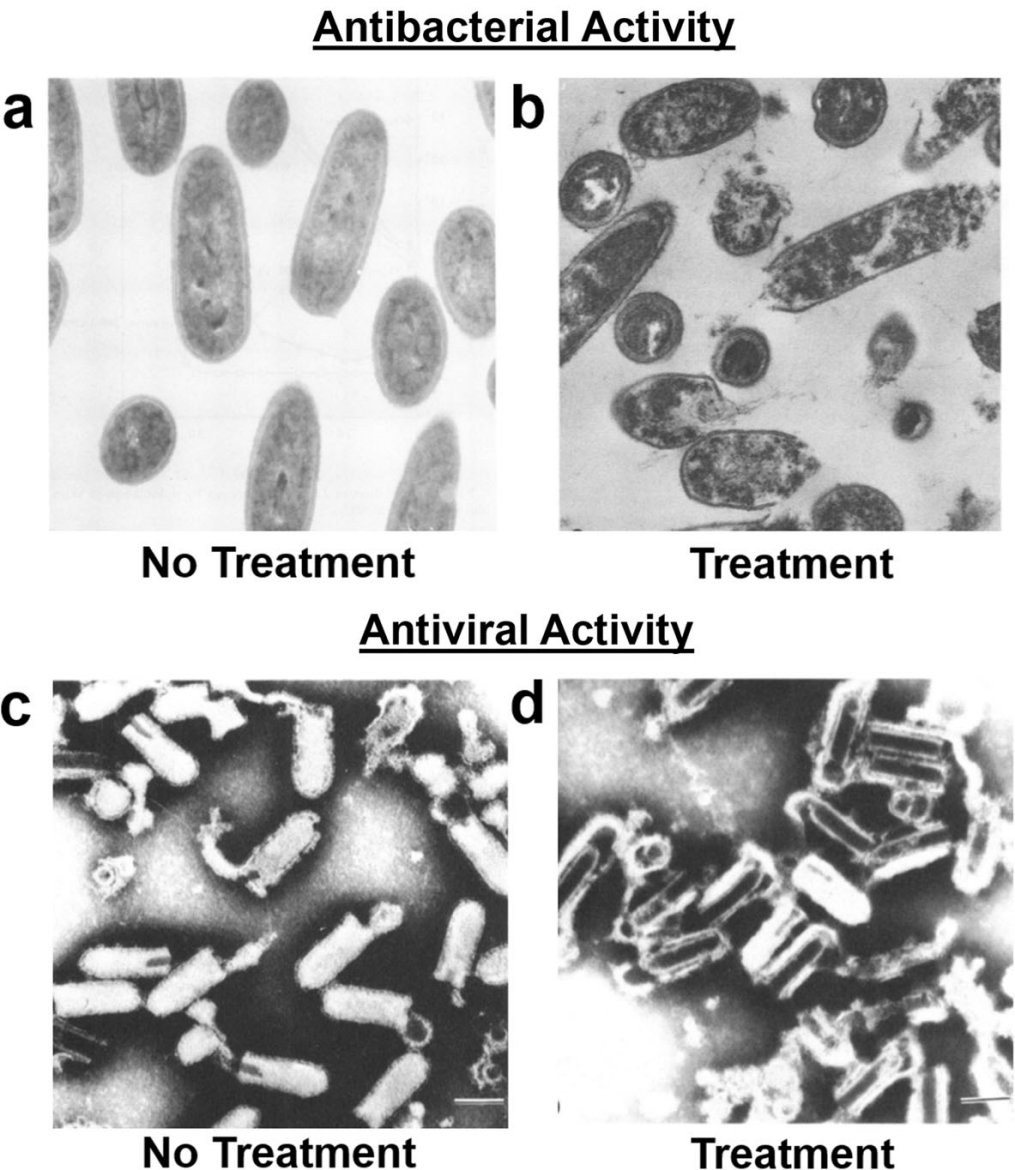
Membrane disruption of bacteria and enveloped viruses by free fatty acids and monoglycerides. **a** and **b** Transmission electron microscopy images of *L. monocytogenes* bacterial cells **a** without treatment and **b** after treatment with glycerol monolaurate. The magnification scale is × 44,080. Images are from Ref. [27] and reproduced with permission from the American Society of Microbiology. **c** and **d** Electron microscopy images of vesicular stomatitis virus particles **c** without treatment and **d** after treatment with long-chain linoleic acid (free fatty acid). Scale bars, 100 nm. Images are from Ref. [28] and reproduced with permission from the American Society of Microbiology

MCFAs and monoglycerides are antimicrobial agents that can disrupt the phospholipid membrane surrounding membrane-enclosed pathogens such as bacteria and lipid bilayer-enveloped viruses. This membrane-disruptive activity is illustrated in Fig. 1. In terms of antibacterial activity, the compounds can inhibit bacterial growth (“bacteriostatic”) or induce bacterial cell lysis and cell killing (“bactericidal”) [29]. In general, MCFAs and monoglycerides exhibit more potent inhibitory activity against Gram-positive bacteria than Gram-negative bacteria because Gram-positive bacteria have simpler, single lipid bilayer cell membrane structures while Gram-negative ones typically have more complex inner and outer membrane structures. They can also disrupt a wide range of lipid bilayer-enveloped viruses by damaging and/or effectively destroying enveloped virus particles and compromising infectivity [30]. MCFAs and monoglycerides principally exhibit antiviral activity by lysing enveloped virus particles (“virucidal”). On the other hand, MCFAs and monoglycerides are inactive against non-enveloped viruses.

MCFAs and monoglycerides have unique mechanisms of disrupting phospholipid membranes and are principally active in the micellar state [31–33]. Monoglycerides form micelles at lower concentrations than MCFAs, which helps to explain why monoglycerides are often more biologically potent than fatty acids and longer compounds also tend to exhibit more potent inhibitory activity than shorter ones within this group. For example, the C_12_ monoglyceride (glycerol monolaurate, abbreviated as GML) has a lower critical micelle concentration (CMC) value (60 μmol/L at pH 7.4) and typically greater potency than both the C_12_ fatty acid (lauric acid; CMC of 900 μmol/L at pH 7.4) and C_10_ monoglyceride (glycerol monocaprate; CMC of 600 μmol/L at pH 7.4) [34, 35].

Another important consequence of MCFAs and monoglycerides targeting pathogenic membranes is that it is more difficult for susceptible pathogens to develop resistance to these compounds. It is generally acknowledged that there is a very high barrier for pathogens to develop resistance to fatty acids and monoglycerides [36, 37].

### Spectrum of antimicrobial activity

MCFAs and monoglycerides exhibit potent inhibitory activity against a wide range of pathogens, including Gram-positive and Gram-negative bacteria, enveloped viruses, algae, fungi, and protozoa. Pioneering work was completed by the Kabara group in the 1970’s and they conducted detailed structure-function studies investigating how hydrocarbon chain length affects antimicrobial activity [38]. In general, capric (C_10_) and lauric (C_12_) acids exhibited the highest potencies among fatty acids while corresponding monoglycerides with equivalent chain lengths were typically even more potent. Nevertheless, it is important to note that different MCFAs and monoglycerides inhibit different spectrums of pathogens with varying potencies, so appropriate selection is required depending on the pathogen(s) being targeted. More empirical information about the targeting spectrum of different MCFAs and monoglycerides is provided in Ref. [29].

In recent years, there has been interest in exploring the inhibitory activity of MCFAs and monoglycerides against pathogens relevant to the swine industry. It has been reported that GML (C_12_ monoglyceride), lauric acid (C_12_) and a mixture of caprylic (C_8_) and capric (C_10_) acids exhibit potent antibacterial activity against numerous bacterial pathogens such as *Escherichia coli*, *Streptococcus suis*, *Salmonella poona* and *Clostridium perfringens*. It has been reported that MCFAs and monoglycerides often exhibit greater antibacterial potency than commonly studied organic acids (e.g., lactic and citric acids) [39]. It has also been determined that compounds within this class, such as GML, can exhibit nearly complete killing of vegetative cells and spores of aerobic *B. anthracis*, *B. subtilis*, and *B. cereus* and anaerobic *Clostridium perfringens* and *Clostridium (Clostridioides) difficile* [40]. Another study has reported that GML and lauric acid also inhibit *Listeria monocytogenes*, which is a foodborne pathogen that can contaminate swine farms [27].

Furthermore, MCFAs and monoglycerides can inhibit numerous types of lipid bilayer-enveloped viruses, including vesicular stomatitis virus, herpes simplex virus, visna virus, respiratory syncytial virus, parainfluenza virus type 2, avian influenza virus, and ASFv [28, 41–43]. More recent studies have demonstrated that they also exhibit strong antiviral activity against other swine-specific viral pathogens, such as porcine reproductive respiratory syndrome virus (PRRSv) and porcine epidemic diarrhea virus (PEDv) which contain lipid bilayer envelopes that are necessary for structural integrity and infectivity [44, 45].

### Immunomodulatory activity

In addition to antimicrobial properties, certain MCFAs and monoglycerides also exhibit immunomodulatory properties. For example, GML is known to affect immune cells, especially T cell lymphocytes, due to membrane interactions linked to cell signaling pathways [46]. Zhang et al. demonstrated that GML treatment can also decrease cytokine production *in vitro* and thus GML exhibits immunosuppressive effects that can be useful for anti-inflammatory applications [47]. It has been suggested that orally administered GML could be useful for reducing gut inflammation *in vivo* [46]. MCFAs, namely caprylic (C_8_), capric (C_10_), and lauric (C_12_) acids, have also been shown to improve the immune responses of a porcine intestinal cell line *in vitro* [48].

In summary, MCFAs and monoglycerides exhibit antimicrobial and immunomodulatory activities. This combination of membrane-related biological functions, plus the high barrier to resistance development and potential to inhibit both bacteria and enveloped viruses, makes them interesting additive candidates for potential feed pathogen mitigation and for improving enteric health in weaned pigs.

## Production phase vulnerabilities

Strategic use of health-promoting feed additives in pig production is closely associated with the state of immune development. When piglets are born, their immune system development is rudimentary [49]. Thus, during the nursing stage, colostrum is a vital source of energy-rich nutrients, maternal antibodies, and growth factors required for glucoregulation and mucosal immune system development, which are needed for piglet survival [50]. Although nursing pigs receive immune protection from their mother, some bacterial and viral pathogens overwhelm this protection (e.g., *E. coli*)*,* while transboundary pathogens that the mother has not been exposed to (e.g., PEDv) cause nearly 100% piglet death until sows have time to develop immunity which they can pass on via colostral antibodies [1].

The subsequent weaning process is stressful for pigs because of sudden changes in temperature, exposure to pathogens without maternal protection, commingling with other litters, oftentimes transportation to distant nurseries, and a complete change in food source and nutrient form. These factors can lead to profound gastrointestinal and immune dysfunctions, and changes in gut histology and biochemistry along with decreases in nutrient digestion and absorption and gut barrier integrity, all of which negatively affect pig health and growth [51]. Weaning is associated with gut inflammation, immune cell infiltration, and increased levels of pro-inflammatory cytokines. A combination of these effects often leads to reduced feed intake (protective effect) and diarrhea [52].

These energy-intensive immune responses can decrease feed efficiency and there is growing recognition of the need to optimize immune health by neither under-stimulating nor over-stimulating immune responses [53]. In addition, because weaned pigs no longer receive sow milk, the gut microbiota continues to mature amidst several weeks of disturbance and change; this maturation aids in pathogen defense and supports the development of the gastrointestinal immune system [54]. Health intervention strategies are related to weaning age and health status of the sow herd source farm (T. Donovan, personal communication, 2019) in order to support piglet gut and/or respiratory health and immune development [55]. Strategically chosen feed or water-delivered additives can boost a pig’s immune response, protect against detrimental histological changes, reduce pathogenic levels throughout the body (especially in gut and lung), support beneficial microbial populations in the gut, and stimulate digestive function [56].

In addition to evolving immunocompetence during different stages of pig production, another concern is understanding how bacterial and viral infections affect pig health and wellbeing and how feed- and/or water-delivered additives might mitigate such effects. For example, recent evidence shows that viral infections such as PEDv have significant effects on gut microbiota and immunological profiles of pigs. Song et al. discovered that PEDv infection caused significant changes in the gut microbiota of sows and piglets, including decreased abundance of *Bacteroidetes* spp., which is the dominant bacteria in healthy piglets, and increased prevalence of pathogenic bacteria such as *Fusobacteria* and *Proteobacteria* spp. [57]. Huang et al. observed similar effects on the dynamic change of gut microbiota in suckling piglets, with more pronounced changes occurring at later time points during the period of 1- to 3-week of age [58]. Tan et al. also noted similar trends in microbial changes in the cecal mucosa and contents of suckling piglets [59]. In addition, Langel et al. determined that the stage of gestation at which PEDv infection occurs in pregnant sows affects maternal immunity and lactogenic immune protection of neonatal suckling piglets [60]. Other pathogens such as *Salmonella enterica* and *Lawsonia intracellularis* also cause significant changes in the pig gut microbiome [61].

In light of these different production challenges, feed additives such as MCFAs and monoglycerides might play important roles as growth permittants by improving enteric health and by improving the immune response in weaned pigs. The second emerging contribution to animal health is feed pathogen mitigation. The discovery that MCFAs are equally as effective as formaldehyde to prevent the transmission of a feedborne viral pathogen, PEDv, in piglets was a landmark finding [62] and this approach has potential for dealing with other viral and bacterial pathogens as well. These aspects are covered in the remaining sections of this review.

## Growth performance

MCFAs and monoglycerides have emerged as candidates for replacing in-feed antibiotics and promoting animal well-being, including to enhance growth performance. Table 2 summarizes ongoing progress in the field.

**Table 2:** Summary of growth performance results when using medium-chain fatty acids and monoglycerides as feed additives

Test additive	Rate	Development stage	Key findings	Ref.
Caprylic acid/capric acid (6:4 blend)	8%	Weaned pigs at d 21 and evaluated for 28 subsequent days (*n* = 164 pigs)	-FCR was best with fatty acid-containing diet -Decreased serum urea levels, suggesting improved energy utilization -Increased serum triglyceride levels	[63]
Caprylic acid	0.5%	Weaned pigs around d 28–32 and evaluated for 21 subsequent days (*n* = 120 pigs)	-Significantly improved growth performance compared to control group and another group fed 1% triglycerides of capric and caprylic acids -Shedding of *Cryptosporidium* oocysts was delayed and less intense	[64]
Blend of MCFAs (caproic, caprylic, capric, and lauric acids)	0.5%	Piglets around d 7 and evaluated for 77 subsequent days (*n* = 183 pigs)	-Tended to improve overall ADG, with significant differences between days 35–56 -Tended to improve feed efficiency (smaller FCR) -Decreased *Clostridium* levels in the small intestine digesta	[65]
Caprylic acid or capric acid	0.2%	Weaned pigs at d 35 and evaluated for 49 subsequent days (*n* = 252 pigs)	-Improved ADG -Best FCR in pigs receiving caprylic acid-containing diet -Decreased mortality and increased protein and fiber digestibility -Reduced *Clostridium perfringens* levels -Improved mucosal epithelium structure of ileum	[66]
Caproic, caprylic, and capric acids (individual or 1:1:1 blend)	0.25–1.5%	Weaned pigs (7–23 kg body weight) for 35 d (*n* = 360 pigs)	-Improved ADG, AFDI, and FCR with higher MCFA blend dose -Caprylic acid yielded best growth performance among individual MCFAs	[67]
Caproic, caprylic, and capric acids (1:1:1 blend) or one of two blends (12:48:40 or 4:54:38 ratio)	1%	Weaned pigs (6 kg initial body weight) from d 27 for 29 subsequent days^a^ (*n* = 100 pigs)	-Improved FCR in *E. coli*-challenged pigs with MCFA-containing diets -Similar growth performance level of MCFA- or chlortetracycline antibiotic-containing diets	[68]
Lauric acid/GML	0.1%	Weaned pigs during the entire weaning period (7–25 kg body weight) and breeding sows^b^	-Significant reduction in the use of antimicrobials in breeding sows -Daily dose per animal year^c^ was reduced by around 8 d in the test herds	[69]
Caprylic acid and capric acid blend	0.15–0.3%	Growing pigs (28 kg initial body weight) for 35 subsequent days (*n* = 140 pigs)	-Improved ADG (equivalent performance to growth-promoting antibiotic mixture) -Increased lymphocyte percentages and IgG concentrations in pig blood	[70]

^a^Weaning occurred at d 22

^b^Data were reported from 33 test farms which used the feed additive (median of 440 sows per farm) and 29 control farms (median of 685 sows per farm)

^c^Detailed information about the calculation details and a sample calculation are provided in the Material and Methods section of Ref. [69]

In the late 1980s, Cera et al. conducted one of the earliest free MCFA studies in the field, when they measured postweaning growth performance and serum profile responses to supplemental MCFAs [63]. The diet contained 8% MCFA (composed of 60% caprylic acid and 40% capric acid), 8% tallow, or a mixture of fatty acids and tallow (4% each). Over the 28-day test period, the feed conversion ratio (FCR) was most improved for the MCFA containing-diets (*P* < 0.01), along with lower serum urea levels (*P* < 0.05; indicative of improved amino acid utilization) and increased serum triglyceride levels (*P* < 0.05). It was noted that the relatively high fraction of caprylic and capric acids in the feed caused a moderate smell, which could affect palatability. This observation motivated the further exploration of lower doses and exploring more palatable MCFA and monoglyceride options. Such efforts have been further motivated by progress in using 2.5–5% MCT-containing oils or 5% *Cuphea* seeds (a natural MCT source) as feed additives to piglet diets, which resulted in improved growth performance and gut health parameters such as gut microbial composition, anatomical features, and immune profile [13, 14, 71].

Marounek et al. noted positive results when 0.5% caprylic acid was fed to *Cryptosporidium parvum*-infected weaned pigs, as indicated by enhanced growth performance (*P* < 0.05) [64]. Weight gained was 4.97 ± 0.36 kg in the 0.5% caprylic acid-treated group, as compared to 3.64 ± 0.43 kg and 3.95 ± 0.35 kg in untreated control and 1% MCT oil-treated groups, respectively. This finding was important because it showed that inclusion of 0.5% caprylic acid outperformed a 1% MCT oil of caprylic and capric acids in the triglyceride form, thus supporting the utility of free MCFAs as feed additives. There was also a tendency for decreased shedding of *Cryptosporidium* oocysts in treated groups (*P* = 0.096).

Building on this work, Hanczakowska et al. investigated the effects of MCFA supplementation on piglet growth performance and tested 0.5% of an MCFA-containing feed additive mixture that included caproic, caprylic, capric, and lauric acids [65]. The treated group tended to have improved weight gains [average daily gain (ADG) of 268 g vs. 283 g, control and treated groups, respectively; significant changes in ADG during days 35–56 post-birth (*P* < 0.05)] and improved feed efficiency (FCR of 1.48 vs. 1.37, control and treated groups, respectively); *Clostridium* levels tended to decline in digesta of the small intestine, as compared to the control group without additive.

Subsequently, Hanczakowska and colleagues investigated the effect of 0.2% caprylic acid and/or capric acid on weaned piglet growth performance, apparent digestibility of nutrients, intestinal microflora, and ileum structure [66]. ADG was greater (*P* < 0.01) among piglets that received caprylic or capric acids or a combination thereof, as compared to the control group without MCFAs or antibiotics. In addition, mortality decreased and protein (*P* < 0.01) and fiber (*P* < 0.05) digestibility improved for pigs fed MCFAs. Furthermore, *Clostridium perfringens* levels in the ileum were reduced by each type of MCFA (*P* < 0.01), and capric acid caused significant increases in aerobic bacteria (*P* < 0.05) as well. It was also noted that capric acid supplementation led to improvements in the morphology of the mucosal epithelium in the ileum (greater villus height in capric acid-treated vs. control groups; *P* < 0.01).

Gebhardt et al. also evaluated the dietary addition of MCFAs to nursery pig diets for growth promotion [67]. They tested a 1:1:1 blend (weight basis) of caproic, caprylic, and capric acids that were fed at 0, 0.25%, 0.5%, 1.0%, and 1.5% of the diet. Linear dose-dependent improvements in ADG, average daily feed intake (ADFI), and FCR were noted (*P* < 0.01). Although MCFA diets had a strong odor, they did not reduce feed intake. Cochrane et al. further investigated whether MCFAs could be an alternative to the antibiotic chlortetracycline (CTC) in nursery pig diets in a bacterial pathogen challenge model [68]. Pigs were challenged with enterotoxigenic *E. coli*, and fed a diet 1) without MCFAs or CTC, 2) with 0.036% CTC, or 3) with 1% inclusion of a 1:1:1 blend, 4) 12:48:40 blend, or 5) 4:54:38 blend of caproic, caprylic, and capric acids. It was determined that *E. coli*-challenged pigs that received one of the MCFA-containing diets exhibited similar FCR values to those receiving the antibiotic-containing diet.

In addition, De Snoeck et al. investigated the effects of a 0.1% lauric acid/GML feed additive in the diets of weaning piglets on antibiotic use in farrow-to-finish pig farms [69]. In comparison to farms where piglets were only given regular feed without additive, the farms where piglets received the feed additive used eight fewer daily doses of antibiotic per animal year (*P* < 0.01). While most studies have focused on how MCFAs and monoglycerides can support early stages of pig growth and development, Zhang et al. have also investigated the effects of adding 0.15% or 0.3% total MCFA doses (containing 58% caprylic and capric acids plus silica carrier; equivalent to effective total MCFA doses of ~ 0.087% and ~ 0.17%, respectively) to the diet of finishing pigs on growth, nutrient digestibility, and blood profiles [70]. The 0.3% total MCFA supplement led to improved ADG (*P* < 0.05; similar performance to that of a 0.1% antibiotic cocktail of CTC, sulfonamide dimethazine, and procaine benzylpenicillin in a 1:1:1 ratio) and greater lymphocyte percentages (*P* < 0.05) and IgG concentrations (*P* < 0.05) in the blood of MCFA-treated pigs, as compared to the control and antibiotic-treated pigs. No changes in nutrient digestibility were observed. In addition to MCFAs and monoglycerides alone, MCFA additives have been tested in combination with other additives such as organic acids and probiotics.

### MCFA and organic acid mixtures

Hanczakowska et al. investigated the effects of adding 1.5% fumaric acid together with 0.2% of either caprylic acid or capric acid to the diet of weaning piglets. Fumaric/caprylic acid supplementation led to the largest body weight gains; ADG was 276 g vs. 234 g in the untreated control group without additive (*P* < 0.01) [72]. All treatments led to significant decreases in *Escherichia coli* levels in the digesta collected from the small intestine relative to the untreated control group (*P* < 0.01). Hanczakowska et al. also investigated the effects of adding 0.5% propionic and formic acids (1:1 ratio) together with 0.2% of either caprylic acid or capric acid or a combination thereof to the diet of weaning piglets [73]. Compared to the untreated control group, piglets receiving MCFA supplementation had the highest ADG (*P* < 0.05) and improved protein and fiber digestibility (*P* < 0.05), with no difference in FCR.

Upadhaya et al. further investigated how a combination of organic acids and MCFAs affects the growth performance of finishing pigs [74]. A matrix-coated acid supplement was tested that included the following ingredients: 17% fumaric acid, 13% citric acid, 10% malic acid, and 1.2% capric and caprylic acids. As such, the dose of MCFAs (caprylic and capric acids) was very low and the supplement was added at 0.1% or 0.2% of the diet (equivalent to ~ 0.0012% to ~ 0.0024% total MCFA in the diet). It was observed that the acid supplement exhibited dose-dependent effects and improved ADG (*P* < 0.001), increased nutrient digestibility (*P* < 0.001), and caused changes in fecal bacterial populations, as compared to an untreated control group. In particular, there were significant decreases in *E. coli* counts (*P* < 0.001) along with a tendency towards increased *Lactobacillus* counts (*P* = 0.06). In a separate study, Nguyen et al. also tested the effects of adding 0.1% or 0.2% of the same additive mixture on the growth performance of finishing pigs [75]. Increased ADG (*P* < 0.05) was noted in the treated group along with improved feed efficiency (decreased FCR; *P* < 0.05), greater IgG antibody concentrations (*P* < 0.05), and altered bacterial populations (increased *Lactobacillus* and decreased *E. coli* counts; *P* < 0.05), as compared to an untreated control group.

In follow-up work, Upadhaya et al. investigated how a combination of 40% organic acids (17% fumaric acid, 13% citric acid, 10% malic acid), 1.2% MCFAs (capric and caprylic acids), and 58.8% vegetable oil carrier affects the growth performance of weaning pigs [76]. The feed supplement was incorporated into the diet at 0.1% or 0.2%, and inclusion at the 0.2% dose led to improvements in ADG (*P* < 0.05), ADFI (*P* < 0.05), FCR (*P* < 0.05), and nutrient digestibility (*P* < 0.05), as compared to an untreated control group. Lan and Kim also investigated how a combination of 40% organic acids (17% fumaric acid, 13% citric acid, 10% malic acid), 1.2% capric acid, and 1.2% caprylic acid along with 57.6% Kaolin (2SiO_2_·Al_2_O_3_·2H_2_O) aluminum silicate carrier affects the performance of sows and their piglets [77]. The results showed that 0.2% feed supplement did not affect indicators of reproductive performance, however, it did improve piglet growth (ADG; *P* < 0.05) and the microbiota of sows and piglets, namely fecal *Lactobacillus* counts increased (*P* < 0.05) and *E. coli* counts decreased (*P* < 0.05) in sows and piglets.

In another study, Devi et al. incorporated 0.1% or 0.2% of a feed supplement, which contained 17% fumaric acid, 13% citric acid, 10% malic acid, and 1.2% capric and caprylic acids (in a 1:1 mixture), into sow diets and analyzed the effects on lactating sows and their piglets [78]. The incorporation of 0.2% feed supplement led to improved digestibility (*P* < 0.05) in lactating sows along with higher white blood cell and lymphocyte concentrations in suckling piglets (*P* < 0.05), as compared to an untreated control group without additive. Fecal samples showed higher *Lactobacillus* levels and lower *E. coli* levels for both treatment levels (*P* < 0.05). In subsequent work, Lei et al. found that 0.2–0.4% of the same feed additive also improved growth performance of *E. coli*-challenged piglets [79]. There was a protective effect for infected piglets, as indicated by greater body weight gain, daily feed intake, and FCR (*P* < 0.05) compared to an untreated control group without additive. Lower incidence of diarrhea (*P* < 0.05) was also reported.

Kuang et al. also tested the effects of dietary combinations of organic acids and MCFAs as a replacement for zinc oxide on growth, digestibility, and immunity of weaned pigs [80]. A mixture of MCFAs (including lauric and capric acids) plus calcium formate, calcium lactate, and citric acid was incorporated into the diet at 0.3% (equivalent to ~ 0.04% MCFA), and compared to a control diet that included 0.002% colistin sulfate, 0.002% enramycin, and 0.25% zinc oxide. The MCFA-containing mixture improved ADG (293 g vs. 318 g, control and treated groups, respectively; *P* < 0.05) and FCR (2.19 vs. 1.88, control and treated groups, respectively; *P* < 0.05) in pigs weaned at 21 d. This result was not observed in pigs weaned at 28 d. Organic acid/fatty acid supplementation also improved amino acid digestibility (*P* < 0.01), lowered inflammatory immune responses (TNF-α; *P* < 0.05), and increased ileal *Lactobacillus* content (*P* < 0.05).

### MCFA and probiotic mixtures

Aside from mixing MCFAs with organic acids, there have been attempts to combine them with probiotics as a means to ‘seed’ the gastrointestinal tract with beneficial microbiota. Hanczakowska et al. investigated the effect of MCFAs and/or a probiotic (*Enterococcus faecium*) supplement on weaning piglet performance [81]. The diets contained 0.3% caprylic or capric acid either with or without the probiotic (0.35 × 10^9^ colony-forming units of viable bacteria per kg of feed). It was determined that capric acid supplementation improved ADG during the first 70 d of life (*P* < 0.05), as compared to both the control and caprylic acid groups, while tending to reduce piglet mortality and also led to decreases in the length of the jejunum (*P* < 0.05) and mass of the cecum (*P* < 0.05) compared to the control group without additive. Both caprylic acid and capric acid exhibited antibacterial activity against *E. coli* (*P* < 0.05), while inclusion of the probiotic further enabled inhibition of *Clostridium perfringens* in the cecum and jejunum (*P* < 0.01).

Devi and Kim also investigated the effects of adding 0.2% of a palm kernel oil-derived MCFA mixture, which consisted of caproic, caprylic, capric, and lauric acids (equivalent to ~ 0.12% total MCFAs), without or with an additional 0.01% probiotic (*Enterococcus faecium*), on the growth performance of weaned pigs [82]. The probiotic plus MCFAs led to improved ADG and FCR (*P* < 0.05), along with increased plasma glucose levels and nutrient digestibility (*P* < 0.05), as compared to the control group without additive.

Lei et al. also investigated how a blend of matrix-coated organic acids and MCFAs, without or with a probiotic (*Enterococcus faecium*), affected the growth performance of finishing pigs [83]. The feed supplement mixture contained 17% fumaric acid, 13% citric acid, 10% malic acid, 0.6% capric acid, and 0.6% caprylic acid, and the mixture was incorporated at 0.05% or 0.1% of the diet (equivalent to ~ 0.005–0.01% total MCFAs), without or with 0.002% probiotic. Together, the organic acids, MCFAs, and probiotic led to the best growth performance, as indicated by improved ADG, ADFI, and nutrient digestibility (*P* < 0.05). At the same time, there were negligible differences in blood parameters. Altogether, the findings demonstrate that MCFAs, by themselves and in combination with other additives, can exhibit positive effects on growth performance during different stages of the pig production process, an outcome that is likely related in part to improved gastrointestinal tract health.

## Anti-infective strategies

Another emerging area of opportunity is exploring the feasibility of MCFAs and monoglycerides to replace antibiotics and address infectious disease challenges in farm settings. So far, there has been interest in testing whether MCFA additives can curb *Salmonella* infections among pigs. Towards this goal, Messens et al. developed an *in vitro* continuous culture system to simulate the porcine cecum and reported that 15 mmol/L sodium caprylate (the salt form of caprylic acid) could reduce coliform and *Salmonella* counts by > 99.99% (*P* < 0.01) [17].

Rasschaert et al. investigated the effect of MCFAs on reducing *Salmonella* shedding and colonization in slaughter age pigs on a farm with high *Salmonella* prevalence [84]. A feed additive mixture composed of MCFAs (including caproic, capric, caprylic, and lauric acids), lactic acid, and oregano oil was incorporated into the diet at 0.371%, and compared to diets containing butyric acid alone (0.13% of feed), a mixture of short-chain organic acids (0.292% of feed), and a control group without additive. Among the tested additives, only the MCFA-containing additive mixture was able to reduce *Salmonella* levels in pre-slaughter feces (*P* < 0.01), in cecal contents (*P* < 0.05), and in the ileocecal lymph nodes (*P* < 0.01) after slaughter, as compared to the negative control group without additive. Thus, the feed additive was able to reduce *Salmonella* prevalence by around 50% and decreased *Salmonella* levels in cecal contents and in the lymph nodes by around 36% and 67%, respectively.

In addition, Ren et al. evaluated the protective effect of a mixture of organic acids and capric acid on the inflammatory immune response of *E. coli*-challenged piglets [85]. The mixture was incorporated into the diet at 0.64% formic acid, 0.25% propionic acid, and 0.2% capric acid. The treated group had reduced inflammatory responses, as indicated by decreased concentrations of plasma tumor necrosis factor-α (*P* < 0.05) and interferon-γ (*P* < 0.05) at 9-h and 24-h post-challenge, along with reduced incidence of diarrhea.

Recently, López-Colom et al. also investigated the efficacy of an MCFA salt-containing feed additive against two enteric pathogenic challenges, *Salmonella* Typhimurium and enterotoxigenic *E. coli*, in weanling piglets [86]. The additive consisted of a coconut oil distillate and contained 32.4% lauric acid, 4.2% caprylic acid, and 3.9% capric acid along with other longer-chain fatty acids. The feed additive was incorporated into the diet at 3% (equivalent to ~ 1.1% lauric acid). In both challenge models, antibacterial effects were observed in the hindgut, as indicated by reduced *Salmonella* spp. levels in the cecum (*P* = 0.03; *Salmonella* trial) and a tendency towards decreased enterobacteria and total coliform levels in the ileum and colon (*P* < 0.10; *E. coli* trial). The feed additive also tended to modulate colonic microbiota in both models, while there were no marked changes in intestinal fermentation products, serum pro-inflammatory mediators, or histological parameters.

Together, the data support that MCFAs can at least curb certain types of bacterial infections in pigs and additional investigations are warranted, especially against viral infections such as PEDv and ASFv.

## Gut function and immune health

The gastrointestinal tract plays an important role in facilitating nutrient digestion, absorption, and metabolism along with critical immune functions. It has been reported that MCFAs and monoglycerides can improve immune health-related parameters and such findings have motivated deeper investigation of the mechanistic underpinnings.

**Fig. 2:**
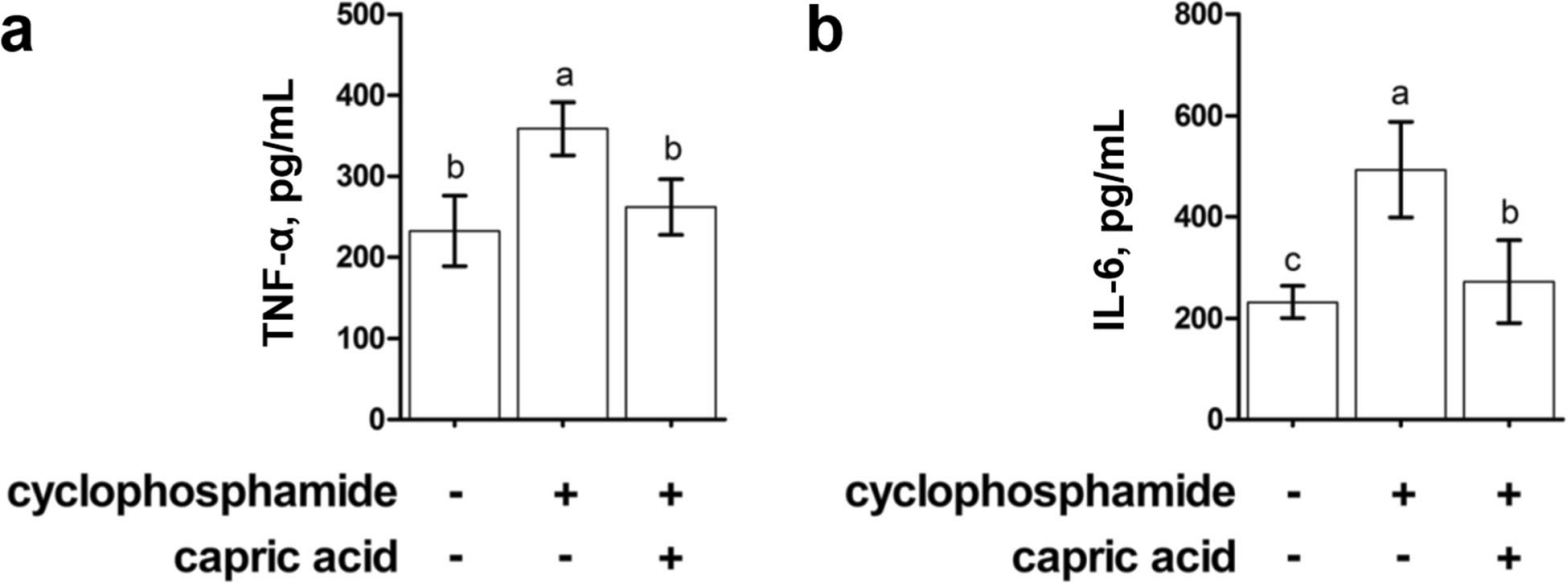
Anti-inflammatory effects of capric acid feed additive in immunosuppressed miniature pigs. The levels of **a** TNF-α and **b** IL-6 pro-inflammatory cytokines in serum were measured by enzyme-linked immunosorbent assay (ELISA). Cyclophosphamide (−) and capric acid (−) indicates the negative control group (immunocompetent pigs), cyclophosphamide (+) and capric acid (−) indicates immunosuppressed pigs that were not given capric acid feed additive, and cyclophosphamide (+) and capric acid (+) indicates immunosuppressed pigs that were given capric acid feed additive. Data are from Ref. [87] and reproduced with adaptation under the terms of the Creative Commons CC BY license

Lee and Kang studied the effects of 0.5% capric acid on protecting miniature pigs against cyclophosphamide-induced intestinal inflammation, oxidative stress, and gut barrier function, whereby cyclophosphamide acts as an immune suppressant [87]. It was determined that capric acid protected against inflammatory cytokine production while tempering oxidative stress and aiding intestinal barrier function *in vivo* (Fig. 2). Wang et al. have also shown that caprylic acid can increase the expression levels of endogenous host defense peptides, such as β-defensins, in order to enhance intestinal epithelial barrier function in an *in vitro* cellular model [88, 89].

In addition, Ferrara et al. investigated how short-chain organic acids and MCFAs affect gut morphology and intestinal immune cells in weaned piglets [90]. The authors tested a combination of 0.15% caprylic and capric acids plus 0.41% fumaric acid and 0.32% lactic acid as well as the organic acid mixture alone. It was determined that the organic acid mixture increased the number of intra-epithelial lymphocytes in the jejunum, and similar results were obtained when the MCFAs were added together with the organic acid mixture.

There has also been interest in understanding how MCFA additives affect gut-related microbial populations, which are intimately linked with proper immune function. For example, Zentek et al. investigated how MCFAs (~ 0.15% caprylic and capric acids in total) affect gastrointestinal microbial ecology and bacterial metabolites in the digestive tract of weaning piglets [91]. Notably, there were increased cell counts for certain types of bacteria in the gastric contents, including eubacteria (*P* < 0.001), *Enterobacteriaceae* (*P* < 0.001), clostridial clusters I (*P* < 0.001) and IV (*P* = 0.019), *Lactobacillus johnsonii* (*P* < 0.001), and *Lactobacillus amylovorus* (*P* < 0.001).

## Delivery strategies

While MCFAs and monoglycerides have proven to be useful feed additives, there has also been exploration of different delivery strategies to encapsulate them in order to slow down absorption, guide delivery to targeted regions of the gastrointestinal tract, and thus potentially enhance functional performance. One effective strategy involves using MCTs as the feed additive because they protect the MCFAs and monoglycerides from rapid absorption while the active compounds can be gradually released by lipases *in vivo* [14, 71, 92].

Another emerging approach involves delivering free MCFAs and monoglycerides that are either coated with a protective layer or encapsulated within a carrier. For example, Boyen et al. investigated whether a 0.31% dose of coated caprylic acid (appeared to be fat-coated calcium caprylate) could decrease fecal shedding of *Salmonella typhimurium* in infected piglets [93]. At 2 d post-infection, fecal shedding in the treated group of piglets tended to decrease by about 90% compared to the untreated group. On the other hand, treatment with an equivalent dose of uncoated caprylic acid had negligible effect, pointing to the likely importance of developing an effective delivery strategy.

Zentek et al. further examined the distribution of caprylic and capric acids, either uncoated or coated with vegetable fat and lecithin, within the digestive tract of piglets and resulting effects on gut microbiota and bacterial metabolic products [91]. The piglets were fed a control diet without additive or diet with ~ 0.3% MCFA supplement (~ 0.15% of caprylic and capric acids plus silicon dioxide and other excipients, either aromatic substances or vegetable fat and lecithin) and were then harvested after 4 weeks in order to measure the concentration of fatty acids in the stomach and various sections of the gastrointestinal tract. MCFA concentrations were significantly higher (*P* ≤ 0.001) in the stomachs of piglets receiving an MCFA supplement, although the highest concentrations were only present at the beginning of the gastrointestinal tract due to efficient absorption. There was no significant difference in the results obtained for uncoated or coated MCFA supplements. In addition, dietary supplementation with uncoated or coated MCFA similarly influenced bacterial populations in the stomach, as indicated by the aforementioned increases in the cell numbers of eubacteria and *Enterobacteriaceae*, along with increased ammonia concentrations in the distal small intestine (*P* < 0.001). The authors also noted that coated MCFA supplementation tended to cause the most distinct changes in gut microbiota, although further investigation is warranted to determine whether that effect is due to the coating material itself or due to slower release of the fatty acids.

In addition, Han et al. investigated the use of a micro-encapsulated eucalyptus-MCFA dry powder feed additive (supplemented at ~ 0.1% of the diet) to promote piglet growth [94]. The powder consisted of a mixture of caprylic and capric acids plus eucalyptus extract (1:1:2 blend of the three ingredients; equivalent to 0.025% MCFAs) that was micronized and then coated with palm oil before being diluted with a carrier of calcium carbonate, rice husk powder, and wheat powder; calcium carbonate is often incorporated into formulations to improve compound solubility [95]. Significant growth performance was achieved with the eucalyptus-MCFA mixture as compared to the control diet (*P* < 0.05), and it demonstrated similar performance levels to that of an in-feed antibiotic blend (0.003% tiamulin and 0.004% lincomycin) or 0.15–0.25% zinc oxide. Nutrient digestibility was also highest in the group that was fed the eucalyptus-MCFA mixture (*P* < 0.05). Recently, Omonijo et al. developed microparticles that are intended for effective delivery of essential oils and MCFAs to the pig intestinal tract [96]. Thymol and lauric acid were selected as a model essential oil and fatty acid, respectively, and the microparticles were composed of starch and alginate. Depending on the microparticle composition (0–2% alginate), it was possible to control the release profile in simulated intestinal fluid. The synthesized microparticles also exhibited stability under storage at room temperature. Such microparticles, along with other encapsulation technologies such as mesoporous silica nanoparticles [97], warrant further investigation as carriers to deliver feed additives.

In another example, Giorgi et al. investigated whether laurate calcium soap – a saponified version of lauric acid with calcium – could improve the growth performance and health of post-weaning pigs [98]. The motivation for this approach was to slow down lauric acid absorption and thus increase its effective concentration in the upper gastrointestinal tract. The level of laurate calcium soap was ~ 1% of the diet. It was determined that the laurate calcium soap improved feed efficiency by 19% (*P* < 0.01) and reduced mortality, as compared to the control group without additive. There was also improved total antioxidant capacity (*P* < 0.01) and reduced oxidative stress biomarker levels (*P* < 0.01) compared to pigs receiving the control diet and pigs receiving a diet with 0.04% antibiotic (Amoxicillin) [99].

## Feed pathogen mitigation

Feed is known to be a vector that is capable of delivering infective levels of some, but not all, pathogens (e.g., PEDv). Thus, when MCFAs and monoglycerides are delivered as feed additives, they can also play an important role in feed pathogen mitigation by inhibiting infectious pathogens, such as viruses and bacteria, that might be present in the feed and would otherwise remain viable in the feed matrix for extended periods of time [23]. In effect, MCFAs and monoglycerides can potentially decrease the concentration of infectious pathogens in feed and reduce the probability that animals consuming pathogen-contaminated feed become infected.

A prominent example is PEDv. Dee et al. investigated the effectiveness of a 2% MCFA blend of caproic, caprylic, and capric acids (1:1:1 ratio) to inhibit PEDv contamination of various classes of swine feed ingredients [62]. It was determined that the MCFA blend reduced mean PEDv viral loads in the feed ingredients, as indicated by viral RNA concentrations (genome copies) relative to the levels found in the negative control groups treated only with saline solution (*P* < 0.05). Subsequent inoculation of piglets with PEDv-contaminated choline chloride ingredient caused infection, as indicated by detectable PEDv in the small intestine, viral shedding in feces, mild diarrhea, and anatomical changes. By contrast, all piglets inoculated with MCFA-treated, PEDV-contaminated feed ingredients showed no evidence of PEDv infection and the MCFA blend performed equally as well as formaldehyde in the piglet inoculation studies. In another study, Cochrane et al. investigated the effects of including 2% of the same MCFA blend as an additive in four different feed matrices – feather meal, avian blood meal, porcine meat and bone meal, and poultry by-product meal – to inhibit *Salmonella* Typhimurium [100]. It was demonstrated that the MCFA additive inhibited the number of viable *Salmonella* colonies by > 99% compared to the negative control group without treatment (*P* < 0.05) and demonstrated similar performance to a formaldehyde-treated test group.

Cochrane et al. have also systematically studied the mitigating effects of individual types of MCFAs and combinations thereof on PEDv-contaminated feed samples [101]. Test samples included 1% MCFA blend [caproic, caprylic, and capric acids; 1:1:1 ratio] (aerosolized), 1% MCFA blend [caproic, caprylic, and capric acids; 1:1:1 ratio] (non-aerosolized), 0.66% caproic acid, 0.66% caprylic acid, 0.66% capric acid, 0.66% lauric acid, and 1% capric and lauric acid mixture (1:1 ratio). It was determined that the 1% MCFA blends inhibited PEDv to the greatest extent along with caproic, caprylic, and capric acids alone to varying extents (*P* < 0.05). The MCFA feed pathogen mitigation strategy also protected pigs, which consumed PEDv-contaminated feed, against PEDv infection, as indicated by the lack of PEDv in fecal swabs and cecum content. While MCFAs were effective at feed pathogen mitigation, 1–2% amounts of various oils (i.e., canola, coconut, palm kernel, soy) and 0.3% of FRA® C12 Dry (FRA, Raamsdonksveer, Netherlands), the latter of which is composed of GML and additional monoglycerides, did not inhibit PEDv in feed to a significant extent. Coconut oil did, however, delay PEDv infectivity in pigs. Fatty acid profiles of the coconut and palm kernel oils used in the study indicated high fractions of lauric acid (> 45%) while there were appreciably smaller fractions of caprylic and capric acids (5.1% caprylic and 5.2% capric acids in coconut oil; 2.5% caprylic and 2.9% capric acids in palm kernel oil). These data point to the likely importance of including a sufficient amount of C_6_-C_10_ fatty acids for effective feed pathogen mitigation. On the other hand, canola and soy oils did not contain MCFAs and were ineffective.

In related work, Gebhardt et al. also quantified the long-term feed pathogen mitigation effects of MCFAs on PEDv contamination following feed manufacture [67]. The feed samples were prepared with 0, 0.25%, 0.50%, 1.0%, and 1.5% amounts of a MCFA blend [caproic, caprylic, and capric acids; 1:1:1 ratio] along with 0.5% of caproic acid alone, 0.5% of caprylic acid alone, and 0.5% of capric acid alone. The manufactured feed samples were stored in a barn setting for 40 d, before they were intentionally contaminated with PEDv. Viral RNA levels in the feed samples were measured to evaluate mitigation activity. It was determined that the blended mixture along with each type of MCFA alone was effective at mitigating PEDv in the feed (*P* < 0.05), thus demonstrating that the MCFAs appear to remain stable and functionally active once incorporated into the feed.

Altogether, these findings demonstrate that MCFAs can serve as the basis for effective feed pathogen mitigation strategies. Such mitigation strategies might also be useful for preventing transmission of ASFv, which is capable of spreading via feed [102].

## Conclusions and perspective

MCFAs and monoglycerides can be useful for aiding numerous production issues such as growth, pathogen control, gut and immune health, and feed pathogen mitigation, and there is a growing molecular-level basis to explain these performance results. Based on the latest evidence, the data support that MCFAs and monoglycerides are typically useful in the dose range of 0.2–1% for supporting pig growth performance and gut health and in the range of 1–2% for feed pathogen mitigation. These functions are not mutually exclusive and it will be advantageous to find optimal combinations and concentrations of MCFAs and monoglycerides that support both functions. Ultimately, further validation of the results described herein and continued work in this direction are needed through eventual testing in commercial operations, where pig population density within a room and on-site is typically much greater, and where challenge by enteric and/or respiratory pathogens is normally more daunting than in academic facilities. We also see a few additional areas where further work would be beneficial to advance the field, including:

1. Lipid Selection: Most studies concentrate on a relatively narrow range of MCFAs and further exploration of monoglyceride derivatives is warranted, especially since they can be more biologically potent and palatable in some cases.
2. Delivery Strategies: Water-deliverable formulations of MCFAs and monoglycerides (e.g., emulsions; see Ref. [103]) would be useful to develop since water administration can be delivered more quickly than feed. This strategy might help to address immediate needs when infectious disease outbreaks occur.
3. Respiratory Disease Support: We anticipate that medically important antibiotics will continue to be used for treating clinical diseases in pigs, especially respiratory infections [104]. The development of strategic compositions and suitable delivery strategies to enable MCFAs and monoglycerides to reach the lungs would be advantageous to overcome the traditional challenges of antibiotic solutions.

## Data Availability

Not applicable.

## Abbreviations

ADFI: Average daily feed intake
ADG: Average daily gain
ASFv: African Swine Fever virus
FCR: Feed conversion ratio
GML: Glycerol monolaurate
MCFA: Medium-chain fatty acid
PEDv: Porcine epidemic diarrhea virus
PRRSv: Porcine reproductive and respiratory syndrome virus
